# Correction: Serial measurement of cytokines strongly predict COVID-19 outcome

**DOI:** 10.1371/journal.pone.0271141

**Published:** 2022-07-05

**Authors:** Hasan Selcuk Ozger, Resul Karakus, Elif Nazli Kuscu, Umit Emin Bagriacik, Nihan Oruklu, Melek Yaman, Melda Turkoglu, Gonca Erbas, Aysegul Yucel Atak, Esin Senol

The following information is missing from the Funding statement: This article was supported by Gazi University Scientific Research Projects Unit with grant number 5959, code 64/2020-03.
