# Supplementary material for: Serial measurement of cytokines strongly predict COVID-19 outcome
Source: PLoS One. 2021 Dec 2;16(12):e0260623. doi: 10.1371/journal.pone.0260623 (PMC8639000; doi:10.1371/journal.pone.0260623)
Supplement: Attachment — Submitted filename: Answer to reviewers.docx [file pone.0260623.s002.docx]

08.03.2021

Thank you for your interest and valuable contribution to our article. We have revised our article according to your suggestions and made possible changes. We are resubmitting the article by making the changes suggested by the reviewers. New changes are marked in yellow in the manuscript.

Best regards,

Selcuk

Journal requirements

1. Our manuscript re-edited according to PLOS ONE's style requirements.

2. Additional details on participant consent added to the method section.

3. The recommended statement added to the acknowledgment section of our article. The text on funding removed from the manuscript.

4. Ethics other than method section deleted.

Answers to reviewer

1-Thank you very much for your suggestions. Our article is primarily based on the idea that single-measure cytokine analyzes with uncertain sampling time may be misleading due to the dynamic course of cytokines. For this reason, serial evaluations were planned. We think that the analysis on the change values you suggested will contribute to the objectives of the article. Therefore, the current analyzes have been reconsidered by taking into account the changes in cytokinin levels at day 0 and day 3, and necessary changes have been made in the article that we think will meet your recommendations.

2-We agree with you that regression analyzes are necessary to determine the role of cytokines in mortality, regardless of the influence of confounding factors such as, comorbidities, age, etc. However, we think that regression analyzes cannot be performed due to the current sample size especially in the non-survivors group. The number of patients included in the study was limited due to the low number of cases in our country during the study period, the fact that our study was conducted in a single center and central restrictions were imposed on multicenter studies related to COVID-19 in our country. Also, the high cost of financing due to the scope of the study, which aimed to evaluate many cytokines, caused the number of patients included in the study to be limited.This limitation is clearly stated in the limitation section of our article.

3-Based on your suggestions, the study groups were named survivors and non-survivors.

4-The figures in the study were evaluated and created in terms of re-resolution and clarity.
