# Supplementary material for: Serial measurement of cytokines strongly predict COVID-19 outcome
Source: PLoS One. 2021 Dec 2;16(12):e0260623. doi: 10.1371/journal.pone.0260623 (PMC8639000; doi:10.1371/journal.pone.0260623)
Supplement: Attachment — Submitted filename: Answer to reviewers.docx [file pone.0260623.s003.docx]

09.015.2021

Thank you for your interest and valuable contribution to our revised article. We have revised our article according to your suggestions and made possible changes. We are resubmitting the article by making the changes suggested by the reviewers. New changes are marked in yellow in the manuscript.

Best regards,

Selcuk

**Journal requirements**

1.We've reviewed our reference list to make sure it's complete and accurate.

References 23 and 24 ( [24] Costela-Ruiz VJ, Illescas-Montes R, Puerta-Puerta JM, Ruiz C, Melguizo-Rodriguez L. SARS-CoV-2 infection: The role of cytokines in COVID-19 disease. Cytokine Growth Factor Rev. 2020;54:62-75., [25] Petrey AC, Qeadan F, Middleton EA, Pinchuk IV, Campbell RA, Beswick EJ. Cytokine release syndrome in COVID-19: Innate immune, vascular, and platelet pathogenic factors differ in severity of disease and sex. J Leukoc Biol. 2021;109:55-66.) in the first manuscript were omitted from the first revision due to the reorganization of the discussion section. In the current revision, the references were checked and no change was made to the references.

**Answers to reviewer**

1-Thank you very much for your suggestions. The values for IL-1RA in the non-survivor group were corrected to 48 (7.1 - 90.1).

2-You are right, the values for PDGF-AA show normal distribution. For this reason, instead of the median (IQR, 25-75%), it was preferred to presentation as mean ± SD. The error in this presentation was corrected and PDGF-AA values (5011 ± 2884 for non-survivors and 4283 ± 1979 for survivors) were shown.

3-'On the day of admission' was added in the first line of the discussion, according to your suggestion.

4- In our study, the GraphPad (version 9) program was used to create the graphs. Because Figure 1 is a multi-panel graph and many cytokine levels were compared, the desired image resolution could not be achieved. Figure 1 was revised based on your suggestions and your PLOS ONE figure preparation requirements. The pdf format of the graph was checked with the PACE PLOS and GIMP programs. We hope that sufficient quality and resolution have been achieved. But if the problem persists, we can rearrange the graphs by splitting (survivors, survivors, etc.).
